# Herausforderungen der Therapie thorakaler Schmerzen bei zystischer Fibrose (CF)

**DOI:** 10.1007/s00482-021-00603-z

**Published:** 2021-11-11

**Authors:** A. T. Hoffmann, S. Dillenhöfer, T. Lücke, C. Maier, F. Brinkmann

**Affiliations:** Universitätsklinik für Kinder- und Jugendmedizin, Ruhr-Universität Bochum, Alexandrinenstraße 5, 44791 Bochum, Deutschland

**Keywords:** Hypoxie, Epiduralkatheter, Opioide, Nebenwirkung, Kinder, Hypoxia, Epidural catether, Opioids, Adverse event, Children

## Abstract

**Hintergrund:**

Die zystische Fibrose (CF) ist eine multisystemische progrediente Stoffwechselerkrankung mit vorwiegend abdomineller und pulmonaler Beteiligung. Schmerzen sind für Betroffene ein weiteres komplexes und von den Behandlern unterschätztes Problem.

**Methoden:**

Eine Literaturrecherche deutschsprachiger Leitlinien und englischsprachiger Studien zum Thema CF und Schmerzen wurde durchgeführt, zusätzlich die Beobachtungen zur Diagnostik und Therapie eines CF-Patienten mit progredienten thorakalen Schmerzen ausgewertet.

**Ergebnisse:**

Die Recherche ergab, dass zur Diagnostik und Therapie thorakaler Schmerzen bei CF keine deutschsprachigen Leitlinien oder Konsenspapiere existieren. Die europäischen und amerikanischen Erhebungen zeigen aber die große Relevanz des Themas und postulieren einen Zusammenhang von Schmerzintensität mit einer erhöhten Mortalität. Sie enthalten jedoch keine Daten zur Effektivität der Schmerztherapie. Anhand dieser Daten und des Fallberichts eines jungen CF-Patienten mit stärksten Thoraxschmerzen bei pulmonalen Exazerbationen lassen sich die CF-spezifischen Herausforderungen der Schmerztherapie illustrieren. Neben den Schmerzen an sich sind auch Analgetika angesichts der multiplen Organdysfunktionen mit besonderen Risiken wie gastrointestinalen Blutungen, opioidinduzierter Atemdepression oder opioidinduzierter Obstipation verbunden.

**Diskussion:**

Schmerztherapie bei Patienten mit zystischer Fibrose und Multiorganbeteiligung erfordert ein sorgfältiges Monitoring und interdisziplinäres Handeln. Empfehlungen zum Schmerzmanagement sollten in die deutschsprachigen CF-Leitlinien aufgenommen werden.

## Abstract (en)

**Background:**

Cystic fibrosis (CF) is a rare genetic multisystemic disorder with progressive abdominal and pulmonary involvement. Pain is still an underestimated symptom in CF patients.

**Methods:**

A comprehensive review of guidelines and scientific literature on the topic was performed and combined with findings from pain management in a young CF patient with progressive thoracic pain.

**Results:**

German CF guidelines do not cover diagnosis and management of pain in these patients. Studies from Europe and the United States report interactions between intensity of pain and mortality in CF, but do not include data on the efficacy of pain management. These data and clinical observations of a CF patient with episodes of intense thoracic pain are used to illustrate the specific challenges in pain relief.

**Conclusion:**

Pain management in CF requires meticulous monitoring as well as an interdisciplinary approach and should be implemented in the German CF guidelines. The authors also want to suggest recommendations for the treatment of thoracic pain in CF. The range and severity of organ involvement complicates the use both of opioids and non-opioids. Especially opioid treatment carries the risk of hypoxia and opioid-induced constipation (OIC) and needs close medical supervision.

## Hintergrund

Die zystische Fibrose (CF) ist eine seltene genetische multisystemische Stoffwechselerkrankung. Aufgrund zäher Sekrete kommt es zu progredienten inflammatorischen Veränderungen an Pankreas und Lunge. Vor allem die pulmonalen Exazerbationen sind lebensbegrenzend und gehen oft mit ausgeprägten, mit Progredienz der Erkrankung zunehmenden thorakalen Schmerzen einher. Anhand eines exemplarischen Fallberichts sowie der aktuellen Literatur illustrieren wir die Komplexität der Multiorganerkrankung mit intermittierenden Schmerzexazerbationen gefolgt von Phasen der Schmerzfreiheit. Dies erfordert nicht nur eine individualisierte Schmerztherapie, sondern auch Kenntnis und Beachtung diverser krankheitsspezifischer Risiken.

### Multisystemerkrankung zystische Fibrose

Die CF ist die häufigste autosomal-rezessiv vererbte multisystemische Stoffwechselkrankheit in Deutschland (Inzidenz 1:2500–3500; [28]). Mutationen im Cystic-fibrosis-transmembrane-conductance-regulator(CFTR)-Gen führen zu Funktionsstörungen des CFTR-Chloridionenkanals in der Zellmembran diverser exokriner Drüsen mit daraus resultierender Viskositätserhöhung der Sekrete. Dadurch kommt es zur konsekutiven Obstruktion und nachfolgenden Inflammation im Gastrointestinal- und Respirationstrakt (Tab. 1), deren Progression die Lebenserwartung auf derzeit ca. 55 Jahre begrenzt. Ab dem Kindesalter können rezidivierende pulmonale Exazerbationen zur Zunahme der pulmonalen Symptomatik (Dyspnoe, Husten, vermehrt zähes, schlecht mobilisierbares Sekret) sowie Müdigkeit und Gewichtsabnahme führen [23, 28]. Betroffene klagen über eine reduzierte Lebensqualität, sozialen Rückzug, verminderte Belastbarkeit und mit dem Alter zunehmend über Schmerzen [28]. Verursacht werden sie akut durch Pleuritiden, Pankreatitiden sowie das distale intestinale Obstruktionssyndrom (DIOS). Osteoporosebedingte Kyphosen oder Arthritiden können chronische Rücken- und Gelenkschmerzen generieren [23, 28]. Schmerzen sind somit Folge und zugleich Indikatoren der Progression der Erkrankung und verstärken die körperliche und seelische Belastung.

**Table 1:** 

CF-bedingte Organdysfunktionen und Komplikationen (Auszug)	Pathomechanismus	Symptomatische Behandlung	Häufigkeit
Pulmonale Exazerbation, Folgen: obstruktive und restriktive Ventilationsstörung, Bronchialwandverdickungen, Bronchiektasien, Atelektasen, Bullae	Sekretverlegung, Inflammation, gestörte mukoziliäre Clearance, Infektion/Keimbesiedlung mit Problemkeimen (z. B. *Pseudomonas aeruginosa* [PsA])	Inhalation mit Antibiotika, hypertoner Kochsalzlösung, Dornase alpha; Atemphysiotherapie, resistogrammgerechte i.v.-Antibiose Ultima Ratio: Lungentransplantation	Kinder ab 10. LJ: 30 % PsA-positiv Erw.: 80 % PsA-positiv
Allerg. bronchopulmonale Aspergillose (ABPA)	Immunologisch-entzündliche Reaktion auf *Aspergillus-fumigatus*-Antigene, Kombination allerg. Reaktion Typ I und Typ III/IV	Repetitive systemische Steroidpulstherapie	2–15 %
Exokrine Pankreasinsuffizienz mit Maldigestion, Gedeihstörung und Vitaminmangel	Sekretverlegung, Pankreasatrophie/-fibrose	Pankreasenzymsubstitution, Vitaminsubstitution, hochkalorische Ernährung	85 %
Pankreatogener Diabetes mellitus	Pankreasfibrose mit endokriner/exokriner Insuffizienz	Orale Antidiabetika, Insulin	12–30 % der Jugendlichen und Erw.
Hepatopathie/Leberzirrhose mit Gefahr der portalen Hypertension, Ösophagusvarizen etc.	Sekretverlegung Gallengang, Gallensteine, Inflammation	Ursodeoxycholsäure, Varizensklerosierung/-ligaturen, Shuntoperationen, Lebertransplantation	Fokal biliäre Zirrhose: Kinder 20–50 %, Erw. bis zu 72 % Multilobuläre Zirrhose: Kinder < 1 %, Erw. bis zu 24 %
Gastroösophagealer Reflux und Ösophagitis	Relaxation Ösophagussphinkter durch Lungenbeteiligung und Medikamente, Zwerchfellrelaxation durch Husten und Atemphysiotherapie	Protonenpumpeninhibitoren längerfristig	25 %
Mekoniumileus	Obstruktion im term. Ileum durch zähes Mekonium	Chirurg. Entlastung	10–20 % der Neugeborenen
Distales intestinales Obstruktionssyndrom (DIOS)	Obstruktion im terminalen Ileum und proximalen Kolon durch eingedickten Darminhalt	Orale Gabe großer Mengen isoosmotischer Polyethylenglykollösungen, ggf. chirurg. Entlastung	15–20 Jahre: 5–7,5 % 20–25 Jahre: bis zu 35 %
Osteopenie/-porose mit Gefahr von Kyphosen und Frakturen	Vitamin-D- und Kalziummangel, Steroidtherapie, Inaktivität	Bisphosphonate, Vitamin-D- und Kalziumsubstitution	–
Arthritiden	Inflammation	NSAR, Steroide	2–8 % ab Jugendalter
Azoospermie/Fertilitätsstörung	Obliteration Ductus deferens	–	> 95 % der Männer
Salzverlustsyndrom	Hoher Salzgehalt im Schweiß, Fieber, Hitze	Orale oder i.v.-Zufuhr der fehlenden Salze	–
Sekundäre Nephropathie	Nephrokalzinose, medikamentös-toxisch, diabetesassoziiert, durch Lungenbeteiligung	Dosisreduktion/Absetzen des toxischen Agens, Optimierung Diabetesbehandlung, Dialyse	Selten
Depression	?	Antidepressiva	Häufig, bei Kindern eher Angststörungen

Die Therapie der CF war jahrzehntelang symptomatisch (Atemphysiotherapie, Inhalationen, Pankreasenzymsubstitution etc.). Seit Kurzem können für bestimmte Mutationen durch CFTR-Modulatoren pulmonale Exazerbationen reduziert und die Lungenfunktion stabilisiert werden [8]. Patienten mit anderen Mutationen können nur symptomatisch behandelt werden.

## Methoden

Es wurde eine umfassende Literaturrecherche in der PubMed-Datenbank zu den Begriffen „cystic fibrosis“ AND „pain“ AND „adults“ OR „children“ AND „treatment“ OR „adverse events“ durchgeführt. Darüber hinaus wurden die deutschsprachigen Zystische Fibrose (CF)-Leitlinien (AWMF) nach Empfehlungen zu Schmerztherapie bei CF durchsucht. Und es wurden klinische Beobachtungen der erwünschten und unerwünschten Wirkungen der analgetischen Therapie eines jungen CF-Patienten mit thorakalen Schmerzen ausgewertet.

## Ergebnisse

### Literaturrecherche zu Schmerzen bei Zystischer Fibrose (CF) und ihrer Behandlung

Die Recherche ergab, dass in Deutschland zur Schmerztherapie bei CF weder Studien noch spezielle Leitlinien existieren. Diverse europäische und amerikanische Erhebungen der letzten zwanzig Jahren zeigen aber, dass Schmerzen ein häufiges und gravierendes Problem für die Betroffenen darstellen und oft inadäquat behandelt werden [23, 28]. Unserer Kenntnis nach gibt es auch international nur ein evidenzbasiertes Konsensuspapier einer Universität zur Behandlung von Schmerzen und Angststörungen bei Erwachsenen mit CF^1^ [20]. Es fehlen weltweit prospektive Studien zur Wirksamkeit und zu Gefahren einer Schmerztherapie bei dieser schweren Systemerkrankung.

Schmerzen bei der CF sind durch die erhöhte Inflammation mit vermehrter Ausschüttung proinflammatorischer Zytokine bedingt [28]. Zudem ist es aber denkbar, dass die Fehlfunktion von CFTR-Chloridkanälen in nozizeptiven Neuronen zu einem verstärkten Schmerzempfinden beitragen könnte [31]. Die Lokalisation der Schmerzen bei der CF variiert interindividuell entsprechend dem Ausmaß der Organaffektion und der Krankheitsdauer (Tab. 2).

**Table 2:** 

Lokalisation	Kinder und Jugendliche [%]	Erwachsene [%]
Abdominelle/viszerale Schmerzen	42–100	19–50
Magenschmerzen	10	10–51
Thoraxschmerzen	10–38	9–72
Rückenschmerzen	6–16	19–70
Kopf- und Nackenschmerzen	13–42	6–64
Gelenkschmerzen	11–19	6–27
Muskuloskeletale Schmerzen	3–19	6–44

Während zunächst Abdominalschmerzen dominieren, treten bei Jugendlichen die Thoraxschmerzen mit zunehmender Häufigkeit von Exazerbationen in den Vordergrund. Zwei Studien zeigten einen Zusammenhang zwischen dem vermehrten Auftreten von Thoraxschmerzen und einem erhöhten Mortalitätsrisiko [14, 28]. Je schlechter die Lungenfunktion ist, desto häufiger treten starke Schmerzen auf [23, 28]. Ursächlich für Thoraxschmerzen können Myalgien sein, zumeist sind es jedoch viszerale Schmerzen im Rahmen von Exazerbationen durch Pleuritiden und Pneumonien. Diese werden in Einzelfällen verkompliziert durch Pneumothorax oder Rippenfrakturen [14, 23, 28].

Die Schmerzen sind meist intermittierend und in ihrer Frequenz bei Kindern und Erwachsenen vergleichbar, bei Letzteren chronifizieren sie häufiger. Auch unter der meist lebenslang notwendigen symptomatischen Therapie (insbesondere autogene Drainage und Atemphysiotherapie) sowie bei der Diagnostik (z. B. Spirometrie) treten starke Schmerzen auf [23]. Langfristig finden sich Depressionen, Angststörungen, sozialer Rückzug und eine reduzierte Lebensqualität [21, 23, 28].

Häufig beschrieben wird das geringe Problembewusstsein der Behandler für die Schmerzen und umgekehrt auch die selten aktive Thematisierung der Schmerzen durch die Patienten. Viele Patienten behandeln sich mit frei verkäuflichen Analgetika selbst. Neben Paracetamol (59 %) nehmen die Patienten nichtsteroidale Antiphlogistika (NSAR; 10 %), Acetylsalicylsäure (5 %) und Spasmolytika ein [23]. Opioide werden im Rahmen von Palliativbehandlungen bei Schmerzen und Dyspnoe zur Symptomkontrolle eingesetzt [10, 28, 32]. Gabapentin wird in den USA in 17 % der Erwachsenen-CF-Zentren verschrieben [32]. Viele Patienten versuchen sich auch in alternativen Strategien und Therapieansätzen [15, 28]. Eine einzige prospektive Pilotstudie zeigte eine Reduktion von Gelenkschmerzen und Angst durch regelmäßige Yogastunden [28]. Eine RCT zur Osteopathie fand einen nichtsignifikanten Rückgang von Thorax- und Rückenschmerzen [15].

### Fallbericht

Wir berichten von einem jungen Erwachsenen (ca. 50 kg) mit genetisch gesicherter Zystischer Fibrose (CF) und zunehmenden Schmerzen im Rahmen der Progression seiner Grunderkrankung.

Im Kindesalter standen bei ihm abdominelle Probleme im Vordergrund: Neben einem einmalig aufgetretenen distalen intestinalen Obstruktionssyndrom (DIOS) litt er an rezidivierenden Gastritiden mit zweimalig nachweisbarem Ulcus duodeni. Damals traten wellenartige gürtelförmig in den Rücken ausstrahlende Oberbauchschmerzen (NRS 7–8/10) auf, die mit Metamizol und Butylscopolamin sowie unregelmäßig mit NSAR behandelt wurden. Ab diesem Alter fiel eine milde Hepatopathie auf.

Seit dem 12. Lebensjahr stieg die Zahl krankenhauspflichtiger pulmonaler Exazerbationen an, seit dem 18. Lebensjahr auf bis zu sechsmal jährlich. Neben vermehrtem Husten und verminderter Belastbarkeit klagte der Patient über zunehmende Dyspnoe. Die Lungenfunktion verschlechterte sich jetzt kontinuierlich, teilweise lag die Einsekundenkapazität (FEV1) unter 20 % (aktuell 45 %).

Es erfolgte stets eine resistogrammgerechte antibiotische Therapie, Atemphysio- und Inhalationstherapien. Bei zusätzlichem Asthma bronchiale erhielt er inhalative und im Intervall systemische Kortikosteroide.

**Figure 1:**
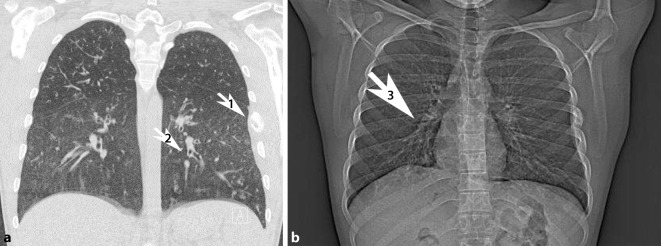

Seit 2 Jahren klagte der Patient im Rahmen seiner Exazerbationen vermehrt nachts sowie beim Husten und bei Inspiration über linksseitige parasternale und paravertebrale als „brennend“ beschriebene, vermutlich überwiegend pleuritisch bedingte Thoraxschmerzen von hoher Intensität (NRS 7–8/10). Die Schmerzintensität war wechselhaft und trat auch bei der Atemphysiotherapie bzw. bei Durchführung einer Spirometrie auf. Ein Pneumothorax wurde stets ausgeschlossen. Im letzten Halbjahr traten pulmonale Exazerbationen mit stärksten Thoraxschmerzen (NRS 7–9/10) alle 2–4 Wochen auf. Die Schmerzintensität war wechselhaft, verstärkt bei Stress und durch Ängste und wurde gemildert bei Ablenkung. In der konkreten Akutsituation ergab sich unseres Ermessens keine Option für eine psychotherapeutische Intervention. Wie zuvor war er in den Wochen und Monaten zwischen den Episoden schmerzfrei. Zuletzt fanden sich in einer CT neben Bronchiektasen und narbigen Residuen zudem bei nachgewiesener Osteopenie unterschiedlich alte Rippenfrakturen (Abb. 1).

Seit ca. 2 Jahren mussten in Akutphasen intravenös appliziertes Metamizol und Paracetamol sowie Ibuprofen immer häufiger durch Piritramid ergänzt werden. Hierunter waren zwar die Schmerzen geringer (NRS 3–5/10), aber der Patient atmete flacher und konnte weniger Sputum mobilisieren. 2020 erfolgte ein Behandlungsversuch mit Gabapentin neben einer festen Analgesie mit Metamizol. Dieses wurde von dem Patienten als wirksam empfunden und daher mehrfach wiederholt. Zusätzlich erfolgten mehrfach manualmedizinische Behandlungen, die nur eine leichte Beschwerdelinderung erbrachten.

Im Herbst 2020 war die beschriebene, bislang in der Klinik übliche Kombinationstherapie nicht mehr ausreichend. Es wurde daraufhin, da in der Vergangenheit mehrfach bei CF-Patienten erfolgreich durchgeführt (s. unten), in Höhe Th7/8 ein Epiduralkatheter angelegt. Darüber wurden Ropivacain und Sufentanil appliziert. Dennoch klagte der Patient weiter über starke Schmerzen, sodass am ersten Tag parallel Piritramid injiziert wurde. Hierunter kam es zu nächtlichen Hypoxien (min. Sauerstoffsättigung 85 %) mit intermittierendem Sauerstoffbedarf von max. 3 l/min über einige Stunden. Am 2. Tag erfolgte eine Umstellung von Piritramid auf Buprenorphin sublingual. Danach traten keine Hypoxien mehr auf, obwohl er bis zum 4. Tag parallel Sufentanil epidural erhielt, danach 3 Tage nur Ropivacain (Liegedauer: 7 Tage).

Zusätzlich entwickelte er rasch eine zunehmende opioidinduzierte Obstipation (OIC) mit beginnendem Subileus und Gefahr eines distalen intestinalen Obstruktionssyndrom (DIOS). Sie konnte durch Absetzen von Piritramid und mehrtägige Naloxegol- sowie Macrogolgabe gebessert werden.

Bei zwei weiteren stationären Aufenthalten gelang mit einer Infusion von Parecoxib sowie einer Epiduralanalgesie mit Sufentanil und Ropivacain eine ausreichende Schmerzkontrolle ohne parallele Gabe systemischer Opioide. Dennoch traten erneut nächtliche Hypoxien auf, die erst nach Absetzen des Sufentanils verschwanden. Nach den Entlassungen beendete er bei Schmerzfreiheit die analgetische Therapie jeweils vollständig.

## Diskussion

**Figure 2:**
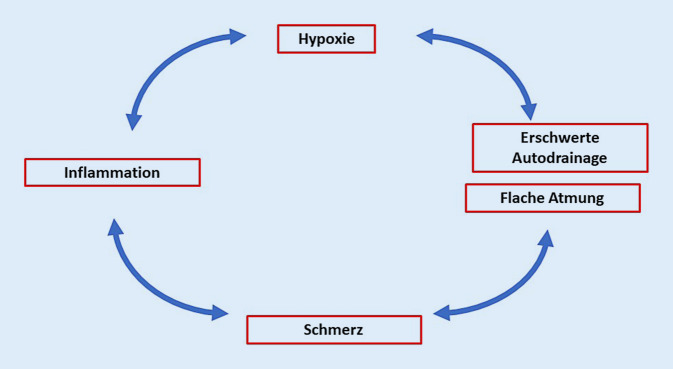

Unsere selbstkritisch intendierte Fallstudie und die Literaturübersicht unterstreichen die Notwendigkeit eines schmerztherapeutischen Konzepts. Sie illustrieren aber auch die Schwierigkeiten bei der Umsetzung. Das Übersehen bestimmter Neben- und Wechselwirkungen der Analgetika kann den Teufelskreis von Schmerz und Hypoxie noch verstärken (Abb. 2). Das Für und Wider der wichtigsten Analgetika fokussiert auf die Therapie des akuten Thoraxschmerzes soll im Folgenden diskutiert werden.

Paracetamol (PCM) sowie Nicht steroidale Antirheumatika (NSAR) und in Deutschland (D) auch Metamizol werden von den meisten Patienten mit Thoraxschmerzen, wie auch im Fallbericht beschrieben, als Basismedikation verwendet [23].

Aufgrund ihrer antiinflammatorischen Wirkung ist der Einsatz von NSAR rational begründbar. Allerdings sind NSAR bei Dauergebrauch und in Kombination mit anderen in der CF-Therapie erforderlichen Medikamenten (z. B. Aminoglykosiden) potenziell nephrotoxisch [26]. Das Ulkusrisiko ist bei CF zusätzlich durch eine Hyperazidität erhöht [19]. NSAR erhöhen das Risiko für GI-Ulzera und obere GI-Blutungen noch einmal deutlich [7]. Auch unser Patient hatte seit seinem 10. Lebensjahr mehrfach Gastritiden und Ulzera, die rückblickend eventuell durch Ibuprofen unterhalten waren und sich unter Protonenpumpeninhibitorengabe besserten. Aufgrund der geringeren GI-Nebenwirkungsrate bei gleicher Wirksamkeit wären Coxibe eine Alternative, wenn auch, wie viele der hier genannten Substanzen, bei Kindern nicht zugelassen [2]. Erste positive Erfahrungen in dem hier vorgestellten Fall mit intravenösem Parecoxib waren ermutigend. Sofern möglich, sollten NSAR, trotz einiger theoretischer Vorteile bzgl. Inflammation und Lungenfunktion [28], zurückhaltend eingesetzt werden, eine Dauereinnahme ist bei der CF kontraindiziert.

Aber auch PCM, ein nur schwach wirksames Analgetikum [17], ist keineswegs nebenwirkungsfrei. Aufgrund von Dosierungsfehlern, akzidenteller Einnahme im Säuglings- und Kleinkindalter sowie Einnahme in suizidaler Absicht ist eine Paracetamolintoxikation die häufigste medikamentöse Ursache eines akuten Leberversagens [17]. Angesichts der Häufigkeit von Hepatopathien bei CF (Tab. 1) muss PCM kritisch gesehen und die Wirksamkeit immer durch Auslassversuche überprüft werden [17]. Wir sahen in diesem Fall in der Exazerbation erhöhte Transaminasen, die sich nach Therapieende stets spontan rückläufig zeigten. Ein Zusammenhang mit der PCM- oder NSAR-Gabe ist nicht auszuschließen.

Das ebenfalls häufig auch bei Kindern in D eingesetzte Metamizol hat im Vergleich ein geringeres gastrointestinales, renales und vermutlich auch hepatisches Risiko [16]. Im vorliegenden Fall wurde es i.v. und oral mit guter Wirksamkeit ohne Komplikationen eingesetzt. Relevante Risiken sind lebensbedrohliche allergische Reaktionen und bei rascher i.v.-Injektion eine Hypotension [1]. Die durch Antikörperbildung ausgelöste Agranulozytose ist selten, kann aber auch bei wiederholter Gabe erstmals ausgelöst werden. Sie ist bei rechtzeitigem Absetzen fast immer reversibel, weshalb präventiv immer eine Blutbildkontrolle zu Beginn und 7 Tage später erfolgen sollte [24].

Gabapentin, das auch unser Patient als wirksam beschrieb, wird in den USA durch fast 20 % der CF-Behandler im Erwachsenenbereich eingesetzt [32]. Gabapentinoide sind eigentlich nur bei neuropathischen Schmerzen wirksam [12]. Bei nozizeptiven Schmerzen anderer Ursache sind sie weitgehend wirkungslos [18]. Sie reduzieren zudem weder den Opioidbedarf noch die Rate opioidassoziierter Nebenwirkungen, im Gegenteil, die Frequenz respiratorischer Komplikation nimmt bei kombiniertem Einsatz zu [5, 18]. Sie können daher nur in Einzelfällen empfohlen werden.

Bei zunehmender Progression der Erkrankung sind, wie auch aus dem Fallbericht ersichtlich, Opioide meist unvermeidlich, in der Palliativphase fast immer geboten [23, 28, 32]. Opioide sollten, wie auch in unserem Falle, so lange wie möglich nur zur Akuttherapie zum Einsatz kommen. Andernfalls sollte auch bei CF-Patienten den Empfehlungen zum Langzeiteinsatz von Opioiden (LONTS 2020) gefolgt werden. Ein Stufenkonzept zum Einsatz von Opioiden, wie es zum Beispiel durch die DGVS für chronisch-entzündliche Darmerkrankungen erarbeitet wurde [27], wäre auch für die Behandlung von Schmerzen bei CF sinnvoll.

Bei unstrittiger analgetischer Effektivität sind vor allem die Dosisfindung und die Wahl des Opioids bei der CF aus zwei Gründen von herausragender Bedeutung.

Das erste Problem ist eine opioidinduzierte Obstipation (OIC), welche bei der CF, wie oben beschrieben, leicht zu einem DIOS bzw. Subileus führen kann (Tab. 1; [28]). Das Risiko ist dosisabhängig und nimmt im Verlauf einer Opioidtherapie aufgrund der fehlenden Toleranzentwicklung zu. Reine µ‑Agonisten wie Morphin (und vermutlich Piritramid) haben ein höheres Risiko als Tapentadol oder Buprenorphin [29]. Eine Prävention mit Laxanzien wie Macrogol sollte immer erfolgen. Bei manifester OIC und nicht praktikabler Dosisreduktion sind „peripherally acting my-opioid receptor antagonists“ (PAMORA) wie Naloxegol wegen ihres kausalen Angriffspunkts indiziert [29]. Auch im vorliegenden Fall verschwanden alle OIC-Symptome unter mehrtägiger Naloxegoltherapie und dem Wechsel auf Buprenorphin in verminderter Opioidtagesdosis.

Das zweite, noch gravierendere Problem jeder Opioidtherapie bei der CF ist das erhöhte Risiko einer Atemdepression, die dosisabhängig bis zum Atemstillstand reichen kann [30]. Opioide in höherer Dosis begünstigen vor allem nächtliche zentrale Hypo- und Apnoen, die vermutlich die erhöhte Mortalität einer chronischen Opioidtherapie auch in Deutschland erklären [13, 22]. Bei CF korreliert die Intensität der Schmerzen und damit auch der Bedarf an Opioiden mit der Verschlechterung der pulmonalen Funktion (Abb. 2), d. h. mit konsekutiver Abnahme der Atemvolumina und des Hustenstoßes sowie mit intermittierenden Hypoxien [28]. Bereits jede Vorgeschichte mit Hypoxien erhöht die respiratorische Sensitivität für Opioide [3]. Auch hier haben Tapentadol und Buprenorphin das geringere Risiko. Letzteres hat bei Erwachsenen das größte therapeutische Fenster [9, 31]. Im hier vorgestellten Fall traten bei guter analgetischer Wirkung der relativ niedrigen Buprenorphindosen keine Hypoxien mehr auf.

Ein weiteres Problem könnten die allerdings kontrovers diskutierten proinflammatorischen Opioideffekte sein. Epidemiologische US-Studien mit chronisch Kranken und postoperativen Patienten zeigen, dass unter Opioiden generell das Risiko für Pneumonien und Pneumokokkeninfektionen erhöht war [11, 25]. Hierfür könnten natürlich auch die nächtlichen Hypoxien bedeutsam sein. Daher sollten, außer in der Endphase der Erkrankung, Opioide bei der CF äußerst zurückhaltend und möglichst nie zur Dauertherapie verwendet werden.

Fallberichte seit Mitte der 1990er zeigen, dass interventionelle Verfahren im Falle stärkster Schmerzen hilfreich sein können [6]. Periphere Verfahren wie Interkostalblockaden oder intrapleurale Injektionen von Lokalanästhetika hätten angesichts des Infektionsrisikos thorakaler Katheter vermutlich Vorteile und wären rückwirkend gesehen vermutlich auch hier risikoärmer gewesen [4, 6]. Aktuelle Empfehlungen hierzu waren auch im anästhesiologischen Schrifttum nicht ersichtlich. Hier besteht u. E. aber dringend Handlungsbedarf, wie der Fallbericht unterstreicht: Die epidurale Opioidgabe führte, begünstigt leider durch fehlende Abstimmung aller Beteiligten, zu erheblichen Hypoxien, die zunächst durch die gleichzeitige systemische Piritramidgabe erklärbar waren, später aber auch bei alleiniger epiduraler Gabe auftraten. Wir empfehlen daher einen Verzicht auf epidurale Opioide, da bei enteraler Gabe z. B. von Buprenorphin evtl. atemdepressive Effekte sofort erkennbar sind und durch sorgfältige Dosistitration vermutlich hätten vermieden werden können.

## Fazit für die Praxis

Schmerzen sind ein relevantes, aber nicht ausreichend berücksichtigtes Problem für Patienten mit Zystischer Fibrose (CF) aller Altersgruppen. Schmerzen erschweren die Therapie und gefährden so die Patienten. Eine falsch gewählte oder unzureichend überwachte Schmerztherapie ist mit erheblichen Risiken verbunden. Zur Integration der Schmerztherapie in die CF-Leitlinien ist ein Stufenkonzept erforderlich. Als nächster Schritt muss die Versorgungssituation auch in Deutschland erfasst werden, um dann gezielt Versorgungslücken zu erkennen, mittels geeigneter Studien zu beseitigen und ein Therapiekonzept zu erarbeiten. Hierzu ist eine bessere Zusammenarbeit von Pädiatern, Pneumologen und Schmerzmedizinern erforderlich.

## Footnotes

1 https://www.ohsu.edu/sites/default/files/2018-12/Cystic-Fibrosis-Pain-and-Anxiety-Management-Evidence-Summary_11-27-18.pdf*.*
